# Comparative Efficacy of Residual Insecticides against the Turkestan Cockroach, *Blatta lateralis*, (Blattodea: Blattidae) on Different Substrates

**DOI:** 10.3390/insects11080477

**Published:** 2020-07-28

**Authors:** Sudip Gaire, Alvaro Romero

**Affiliations:** 1 Department of Entomology, University of Kentucky, Lexington, KY 40546, USA; sgaire@uky.edu; 2 Department of Entomology, Plant Pathology and Weed Science, New Mexico State University, Las Cruces, NM 88003, USA

**Keywords:** peridomestic cockroach, invasive urban pest, insecticide efficacy, dry residues, substrate type

## Abstract

The Turkestan cockroach, *Blatta lateralis* (Walker) is an invasive urban pest prevalent throughout the Southwestern United States. Despite the presence of this cockroach in peridomestic areas, there is limited information on strategies that can be utilized by pest management professionals (PMPs) to effectively manage populations of this pest. We evaluated the efficacy of dry residues of liquid insecticides commonly used for household and structural insect pest control: Tandem (0.10% thiamethoxam, 0.03% lambda-cyhalothrin), Transport GHP (0.05% acetamiprid, 0.06% bifenthrin), Temprid SC (0.10% imidacloprid, 0.05% beta-cyfluthrin), Demand CS (0.06% lambda-cyhalothrin), Talstar P (0.06% bifenthrin), and Phantom (0.5% chlorfenapyr) on three different substrates against Turkestan cockroach nymphs. Except for Phantom and Talstar P, all insecticide formulations killed 100% of the cockroaches on concrete, 89–100% on tile, and 77–100% on wood within 4 days. The rate of cockroach mortality varied according to the substrates to which they were exposed. Temprid SC and Transport GHP killed cockroaches faster on tile than wood. Tandem provided a faster mortality rate than Transport GHP and Temprid SC on concrete. Demand CS and Tandem killed cockroaches at similar rates on the three substrates. This study provides information to guide PMPs in their selection of insecticide formulations for the management of Turkestan cockroach infestations.

## 1. Introduction

The Turkestan cockroach, *Blatta lateralis* (Walker) (Blattodea: Blattidae) is a peridomestic cockroach that inhabits arid and semi-arid areas of the world. It is native to Saudi Arabia, Afghanistan, Uzbekistan, Southern Russia, and some parts of Africa [1,2,3]. In the last few decades, this cockroach species has expanded from their native range to other Asiatic countries and other continents [4]. In the United States, Turkestan cockroaches were first detected in 1978 at a military base in Lathrop, California, and it is believed that initial infestation occurred when military equipment was brought back from Middle East and Asian military bases [5]. Since then, infestations of this cockroach have established in desert regions of most other southern states, such as Arizona [6], New Mexico [7], Texas [8], and Georgia [9]. 

Anecdotal accounts by extension entomologists and pest management professionals (PMPs) indicate that Turkestan cockroaches are the most common outdoor cockroach species in many urban areas in the Southwestern United States. In Southern California, the Turkestan cockroach is a prevalent outdoor nuisance pest occupying similar habitats to that of the Oriental cockroach (*Blatta orientalis*, L.) [10]. Recent studies suggested that Oriental cockroaches are being displaced as major outdoor pests in this area due to the faster development and reproduction of Turkestan cockroaches [11]. A rising concern is the expansion of Turkestan cockroaches to areas outside the southwestern United States through pet stores selling them as a feeder species [11]. The spread of the Turkestan cockroach may also facilitate the co-introduction of the invasive *Herpomyces* fungi that naturally live on the cockroaches and cause plant diseases [12].

**Figure 1:**
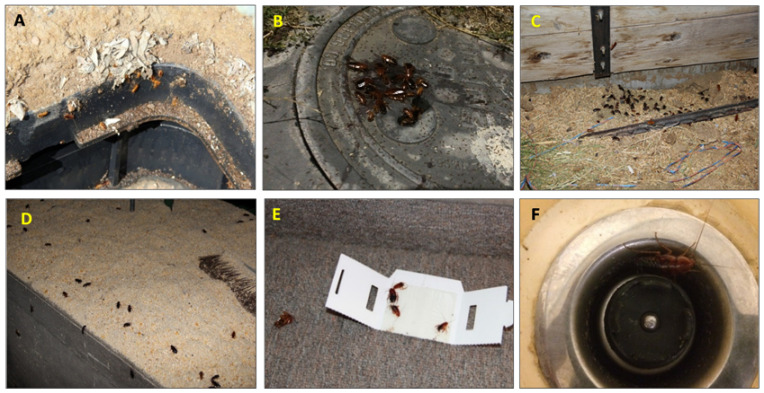
Turkestan cockroaches in outdoor and indoor areas. (**A**) Water meter box, (**B**) manhole, (**C**) sheep feed trough, (**D**) feed mill, (**E**) sticky trap in dorms, and (**F**) kitchen sink.

Turkestan cockroaches live in outdoor areas that offer shelter (e.g., most crevices around structures, sewer system, manholes, crawlspaces, etc.), moisture (e.g., water meter boxes and irrigation boxes), and food (e.g., leaf litter and garbage) (Figure 1) [7]. This cockroach is also found in areas around animal barns where there is organic matter and animal feed (Figure 1). This species has the potential to mechanically vector bacteria such as *Salmonella* spp., a known human and animal pathogen [13,14]. They frequently establish harborages in basements and crawlspaces and become occasional intruders when they disperse from these harborages into private homes, schools, and apartment buildings [7]. 

An effective and environmentally sustainable strategy for the management of peridomestic cockroaches is based on the combination of tactics that include structural exclusion, habitat modification, and a rational use of insecticides through the application of baits and liquid sprays outdoors directly in or near cockroach harborages [15,16,17]. A program that included different management strategies against the smokybrown cockroach, *Periplaneta fuliginosa* (Serville), provided greater control than traditional insecticidal perimeter sprays [15,16]. Integrated management programs that incorporate targeted application of insecticides can reduce human insecticide exposure, the deleterious effects on non-target organisms [18], and the development of insecticide resistance in pest populations [19]. 

Currently, information on integrated pest management (IPM) strategies for controlling Turkestan cockroaches is just beginning to emerge [10,20]. Laboratory and field evaluations conducted by the University of California [10] showed that bait-based programs can be an effective tool for the management of Turkestan cockroaches in environmental sensitive areas. Similarly, Alkan et al. [20] showed that diatomaceous earth (DE) is very effective when used against Turkestan cockroaches. Currently, there is no information available on the residual effect of insecticides on Turkestan cockroaches. Since insecticide efficacy can vary depending on the substrate [21], we evaluated in this study the mortality of Turkestan cockroaches exposed to various insecticide formulations on common harborage materials. The results from this laboratory study might help PMPs select insecticide formulations for targeted application when managing Turkestan cockroach infestations.

## 2. Materials and Methods

### 2.1. Cockroaches

The Turkestan cockroach colony was established from samples collected at New Mexico State University (NMSU) feed mill facility in Las Cruces, NM, in 2014. In the laboratory, colonies were reared at 23–26 °C, a relative humidity of 30–50%, and a photoperiod of 9:15 (L:D) hours. Colonies were reared in plastic containers with egg crates used as shelters. Cockroaches were fed on mixture diet containing rabbit, dog and cat feed as well as sweetened corn puff cereals (1:1:1:1). Water was given regularly. We randomly selected fifth instar nymphs for use in bioassays. Nymphs were used because they were the most abundant life stage of the colony.

### 2.2. Chemicals

Six common commercial insecticides used for household and structural insect pest control were evaluated (Table 1). The chemicals included three neonicotinoids and pyrethroid mixtures (Tandem, Transport GHP, and Temprid SC), two pyrethroids (Demand CS and Talstar P), and one pyrrole (Phantom). 

### 2.3. Substrate

Three substrates: concrete (The QUIKRETE^®^ Companies, Atlanta, GA, USA), pure bond maple unpainted plywood (Columbia Forest Product, Greensboro, NC, USA) and tile (Dal-Tile Corporation, Dallas, TX, USA) were used. Square concrete panels (15.2 × 15.2 cm) were constructed by pouring ready-mix cement into wooden frames and leveling the surfaces. Wood and tile substrates were made by cutting out 15.2 × 15.2 cm^2^.

### 2.4. Residual Bioassay

Each product was diluted with deionized water to the maximum label rate specified by the product label for cockroaches (Table 1). The insecticide solutions were applied at a rate of 40.7 mL/m^2^ (1 gallon/1000 ft^2^) on each substrate using 118 mL fine-mist spray bottles (PRO Chemical & Dye, Fall River, MA, USA). The spray bottles were triggered until they dispensed approximately 0.95 mL (4–5 spray pumps) on each substrate at a distance of approximately 15 cm, resulting in a uniformly treated surface. The amount of active ingredients sprayed per square meter is reported in Table 1. Control squares were sprayed with deionized water in a similar manner. The treated substrates were allowed to dry for 24 h before use.

**Figure 2:**
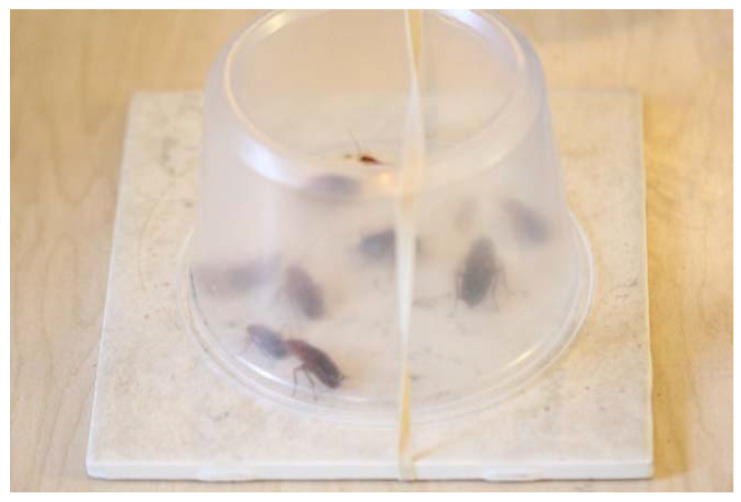
Example of an arena where Turkestan cockroach nymphs were briefly exposed to insecticide-treated substrate. Ten nymphs were introduced in the arena through the opening made by the removal of the bottom of a deli cup. Turkestan cockroaches cannot climb smooth surfaces; therefore, they remained continuously in contact with the treated surface for 5 min.

Groups of nymphs (*n* = 10) were placed on each substrate for 5 min. During this forced exposure period, the insects were confined to the substrate with a deli cup (16 cm diameter, 8 cm height) whose bottom was removed (Figure 2). The deli cup with the cut bottom facing up was secured with a rubber band (Figure 2). After 5 min of exposure to the treated surface, the insects were transferred to insecticide-free deli cups that were maintained in an environmental chamber (24 °C and 40 ± 10% RH) with food and water.

Each treatment was replicated three times. Post-treatment mortality was recorded at 10 min, 30 min, 1 h, 2 h, 4 h, 6 h, 1 day, and daily until day 4. Mortality of cockroaches exposed to Phantom was recorded until day 14. Cockroaches in permanent supine position that did not respond by movements of antennae or extremities upon prodding were classified as dead. 

### 2.5. Statistical Analysis

Time-response mortality data of insecticides on different substrate was analyzed using probit analysis [22,23]. The lethal time necessary to kill 50% of the population (LT_50_) was used to measure the rate at which insecticides caused cockroach mortality. Abbott’s formula was used to correct mortalities [24]. The differences between LT_50_ values of insecticides within substrates were considered statistically significant only when the 95% confidence intervals did not overlap [25]. The method of overlapping of 95% confidence intervals was also used to detect statistically significant differences of LT_50_ values for each insecticide across substrates.

## 3. Results

### 3.1. Comparative Residual Toxicity of Insecticides on Individual Substrates

By day 4, insecticides (except Phantom and Talstar P) killed 77–100% of the Turkestan cockroach nymphs, regardless of the type of substrate used (Table 2). Phantom and Talstar P caused minimal cockroach mortality, which never exceeded 50% by the end of the evaluations. 

Concrete: The amount of time required to kill 50% of the cockroaches (LT_50_) after five-min exposures to insecticides on concrete varied according to the insecticide tested. The LT_50_ for Tandem (LT_50_ = 11.3 h) on concrete was about 50% less than Transport GHP (LT_50_ = 20.4) and Temprid SC (LT_50_ = 19.2 h) (Table 2). There were no statistical significance differences between the LT_50_ values of Transport GHP and Temprid SC or between Transport GHP and Demand CS. There was no mortality observed in the Talstar P treatment, and only 10% mortality in the Phantom treatment (Table 2). Therefore, LT_50_’s could not be calculated for these insecticides on concrete.

Tile: The fastest rate of mortality on tile was observed in cockroaches exposed to Transport GHP (LT_50_ = 10.5 h) (Table 2), and it was statistically significant different from that calculated for Tandem (LT_50_ = 11.3 h). However, no differences in mortality rates were detected between Transport GHP and Temprid SC, or between Transport GHP and Demand CS (Table 2). By day 4, Temprid SC had killed 89% of cockroaches, while Talstar P caused 43% cockroach mortality. Only 20% of cockroaches treated with Phantom were dead by day 14 post-treatment (Table 2).

Wood: Demand CS and Tandem killed 100% of cockroaches by day 4, while exposures to Temprid SC and Transport GHP caused a mortality of 77 and 80% mortality, respectively (Table 2). Cockroaches exposed to Demand CS (LT_50_ = 17.2 h) or to Tandem (LT_50_ = 17.3 h) died significantly faster than cockroaches exposed to Temprid SC (LT_50_ = 28.5 h) (Table 2). Little cockroach mortality was recorded in Phantom (0% mortality) and Talstar P treatments (3% mortality) (Table 2). 

### 3.2. Comparison of Performance of Insecticides across Substrates

Tandem was statistically more effective on concrete than on tile (non-overlapping of 95% confidence intervals of LT_50_ vales) (Table 2), but there were no statistically significant differences between mortality rates of cockroaches exposed to Tandem on concrete and wood (Table 2). Transport GHP provided significantly higher mortality rate of cockroaches exposed on tile than on concrete or on wood (Table 2). Temprid SC performed significantly better on tile than on wood, but no statistically significant differences were detected between mortality rates caused by Temprid SC on tile and concrete, or between concrete and wood. Substrate type did not influence the mortality rate of cockroaches caused by Demand CS (Table 2). Talstar P was moderately effective only on the tile (43% mortality).

## 4. Discussion

Treatments with liquid spray formulations containing insecticides continue to be important for the control of peridomestic cockroaches [26]. Our study is the first laboratory report on the residual efficacy of insecticides against Turkestan cockroaches. We evaluated products containing a pyrrole, and a pyrethroid and/or a neonicotinoid, which are common compounds used by pest control operators for the management of urban pests, including bed bugs, and German cockroaches [17]. The “directions for use” portion of the labels of the products used in our study recommend spot treatments and crack and crevice application in and around surfaces where cockroaches aggregate or have been seen. Turkestan cockroaches often use crevices and cracks of buildings, garages, kitchens, and bathrooms as harborage sites; and common substrates found in these areas include concrete, tile, and wood [7]. Since physical and chemical properties of these substrates can affect the residual toxicity of insecticides [21], we conducted the insecticide exposures on the mentioned surfaces. Similarly, we selected a 5-min exposure to mimic exposure of cockroaches in the field to insecticide deposits when they are arrested in aggregation sites [7]. 

Overall, our results showed that cockroaches exposed to substrates treated with Tandem, Transport GHP, Demand CS, or Temprid SC provided high mortality (≥77–100%) by day 4 post-treatment. At 2-h post-exposure, cockroaches exposed to these products started to show signs of neurotoxicity including excitability, incoordination, and prostration, which are consistent with the neurotoxic effect of pyrethroids and neonicotinoids reported in susceptible insects [27,28]. Cockroaches treated with Talstar P had a low mortality rate (43% on tile). The low efficacy of Talstar P against Turkestan cockroaches may reflect some level of insecticide tolerance that might be the result of continued used of these products for cockroach control at NMSU (David Allan, personal communication). Further studies on insecticide susceptibility of this population and populations from other urban settings with different insecticide exposure history, will be valuable to monitor the development of insecticide resistance in Turkestan cockroaches from areas where this species has become a problem.

We also observed limited mortality of cockroaches exposed to substrates treated with Phantom. This reduced mortality might be explained by its mode of action. The active metabolite of chlorfenapyr (AC 303,268) is a halogenated pyrrole that disrupts the ion transport system of the respiratory chain in mitochondria, resulting in progressive inhibition of adenosine triphosphate synthesis and a delayed lethal effect [29]. The slower effect of Phantom, compared with conventional neurotoxicants, such as pyrethroids, has been observed in the American cockroach, which produced >90% mortality only after 28-day of exposure to dry residues of the product [30]. In light of this, we prolonged the observational period of mortality of cockroaches that were exposed to Phantom to two weeks, but the mortality did not exceed 20% on any of the used substrates. 

Although our results revealed that the majority of tested insecticides provided high efficacy on all tested substrates, the rates at which cockroach mortality occurred varied across different insecticides and substrates. This is indicative of physical and chemical interactions between insecticides and the substrates they were applied on. While the mode of action of the active ingredients, adjuvant and surfactants present in formulations determine the potential insecticidal activity of the compounds [31], the type of substrate greatly affect their residual activity [21]. In general, insecticides on non-porous substrates such as tile, glass, and steel are more effective than on porous surfaces [21,32,33,34]. The effect of substrate type on the insecticide efficacy was evident in our evaluations with Transport GHP and Temprid SC on tile, whose mortality rates were faster when compared with evaluations of the same products on a porous substrate such as wood. Higher mortality rates provided by pyrethroid-based formulations on non-porous substrates has been related to a greater availability of the insecticide deposit to be picked up by the insects [34,35]. The German cockroach, *Blattella germanica* (L.), picked up more permethrin when exposed to glass and tile than plywood and vinyl [32]. On the contrary, more porous materials absorb certain insecticide formulations making them less available to the insects [17] and this seems to have occurred with Transport GHP on concrete and wood, and Temprid SC on wood. A delayed toxicity response of cockroaches treated with Temprid SC and Transport GHP on wood suggest that these insecticide products might be less effective against cockroaches residing on this type of absorptive material. On concrete, the presence of alkaline compounds can accelerate the breakdown of certain insecticides [21,36], and this could have also reduced the toxicity of Transport GHP to Turkestan cockroaches in our study. Some insecticide formulations, however, are not greatly affected by the surfaces they are deposited on [21], and this might have been the case of Tandem and Demand CS, which killed cockroaches at a similar rate on all substrates. Further studies are required to investigate other factors that can affect the toxicity of insecticide deposits to Turkestan cockroaches including the age of insecticide deposits, repellency, and environmental conditions such as humidity and temperature. 

Previous studies have also shown that encapsulated formulations may reduce problems associated with porous and chemically reactive substrates [21,32,37]. We evaluated Demand CS, a product manufactured with microencapsulation technology, which encloses the active ingredient in a polymer shell [35]. These protective effects of microencapsulation seemed to have occurred in the evaluations with Demand CS, which provided similar rates of mortality to cockroaches in all substrates tested. Since Demand CS and Tandem was similarly effective on all three substrates, targeted application of these products might be a useful practice for the management of this pest. 

Selection of proper insecticide formulations based on the substrates found in Turkestan cockroach hiding places, application of baits [10], along with the incorporation of non-chemical methods such as structural modification, exclusion, trapping, physical removal and natural products (e.g., plant essential oils and diatomaceous earth) will increase the chance of a satisfactory reduction of Turkestan cockroach populations [20,38]. 

## 5. Conclusions

This laboratory study compared the efficacy of commonly used insecticide formulations against Turkestan cockroaches on various substrates. All insecticides, except Phantom and Talstar P, produced high mortality in 4 days. Substrate type affected the rate at which some insecticides killed cockroaches. Temprid SC and Transport GHP killed cockroaches faster on tile than wood. Tandem and Demand CS produced a similar mortality rate on concrete, tile, and wood, which suggest that these products can be effective when used against cockroaches harboring on substrates with this type of materials. Our results serve as a baseline for the targeted use of insecticides in management programs for Turkestan cockroach control. Identifying the most prevalent substrates where cockroaches reside is important for selecting the proper insecticide formulation and possibly, the frequency to treat. Successful management of the Turkestan cockroach should be based upon a thorough understanding of their biology and ecology, together with targeted applications of insecticidal baits, sprays, and the inclusion of non-chemical methods.

## Figures and Tables

**Table 1:** Insecticides evaluated in the study.

Trade Name	Formulation	Manufacturer	Concentration of Active Ingredients (AI) in Spray Solution	Amount of AI Sprayed per Meter Square Area (mg/m^2^)
Tandem	Emulsifiable concentration	Syngenta Crop Protection Inc., Greensboro, NC, USA	0.10% thiamethoxam, 0.03% lambda-cyhalothrin	thiamethoxam (40.7) lambda-cyhalothrin (12.21)
Transport GHP	Wettable powder	FMC Corporation, Philadelphia, PA, USA	0.05% acetamiprid, 0.06% bifenthrin	acetamiprid (20.35) bifenthrin (24.42)
Temprid SC	Suspension concentrate	Bayer Crop Science LP, Research Triangle Park, NC, USA	0.10% imidacloprid, 0.05% beta-cyfluthrin	imidacloprid (40.70) beta-cyfluthrin (20.35)
Demand CS	Capsulated suspension	Syngenta Crop Protection Inc., Fulbourn, Cambridge, UK	0.06% lambda cyhalothrin	lambda cyhalothrin (24.42)
Phantom	Suspension concentrate	BASF Corporation, Florham, PA, USA	0.5% chlorfenapyr	chlorfenapyr (203.5)
Talstar P	Suspension concentrate	FMC Corporation, Philadelphia, PA, USA	0.06% bifenthrin	Bifenthrin (24.42)

**Table 2:** Time mortality regression estimates for cockroaches exposed to dry residues of insecticide treatments on various substrates.

Substrates	Insecticides	Lethal Time (LT)_50_^II^, Hours (CI 95%)	Slope ± SE	Percent Mortality ^III^ (%)	χ^2,IV^	df	*p*-Value
Concrete	Tandem	11.3 (9.1–14.0) a	1.49 ± 0.17	100	7.29	8	0.505
	Transport GHP	20.4 (16.3–25.2) b	1.28 ± 0.14	100	6.74	8	0.565
	Temprid SC	19.2 (15.1–24.3) b	1.0 ± 0.10	100	13.43	8	0.098
	Demand CS	15.0 (12.0–18.8) ab	1.19 ± 0.12	100	6.28	8	0.616
	Phantom ^I^	–		10			
	Talstar P ^I^	–		0			
Tile	Tandem	18.1 (14.4–22.5) a	1.26 ± 0.13	100	1.19	8	0.997
	Transport GHP	10.5 (8.30–13.3) b	1.02 ± 0.09	100	3.71	8	0.882
	Temprid SC	12.6 (9.3–17.0) ab	0.62 ± 0.05	89	5.61	8	0.690
	Demand CS	11.3 (8.3–15.5) ab	0.87 ± 0.1	100	6.82	8	0.556
	Phantom ^I^	–		20			
	Talstar P ^I^	–		43			
Wood	Tandem	17.3 (13.9–21.3) ac	1.45 ± 0.16	100	7.67	8	0.466
	Transport GHP	26.7 (20.6–34.7) ab	0.82 ± 0.09	80	3.05	8	0.931
	Temprid SC	28.6 (21.6–38.4) b	0.71 ± 0.07	77	5.79	8	0.670
	Demand CS	17.2 (13.7–21.2) ac	1.45 ± 0.17	100	2.58	8	0.886
	Phantom ^I^	–		0			
	Talstar P ^I^	–		3			

^I^ LT_50_ values for Phantom and Talstar P were not calculated because their mortality level did not exceed 50%. ^II^ Statistical significance based on non-overlap of 95% confidence intervals among insecticide products within substrate. LT_50_ values with the same letter within each substrate are not significantly different (*p* > 0.05; based on the method of overlap of 95% confidence intervals) [23]. ^III^ Percent mortality of cockroaches at 4 d. Mortalities in Phantom evaluations were recorded until day 14. ^IV^ Larger values of χ² for goodness-of-fit and *p* values < 0.05 indicate a poorer fit on the probit regression line.
